# Potential of Best Practice to Reduce Impacts from Oil and Gas Projects in the Amazon

**DOI:** 10.1371/journal.pone.0063022

**Published:** 2013-05-01

**Authors:** Matt Finer, Clinton N. Jenkins, Bill Powers

**Affiliations:** 1 Biodiversity Program, Center for International Environmental Law, Washington D.C., United States of America; 2 Department of Biology, North Carolina State University, Raleigh, North Carolina, United States of America; 3 E-Tech International, Santa Fe, New Mexico, United States of America; University of Florida, United States of America

## Abstract

The western Amazon continues to be an active and controversial zone of hydrocarbon exploration and production. We argue for the urgent need to implement best practices to reduce the negative environmental and social impacts associated with the sector. Here, we present a three-part study aimed at resolving the major obstacles impeding the advancement of best practice in the region. Our focus is on Loreto, Peru, one of the largest and most dynamic hydrocarbon zones in the Amazon. First, we develop a set of specific best practice guidelines to address the lack of clarity surrounding the issue. These guidelines incorporate both engineering-based criteria and key ecological and social factors. Second, we provide a detailed analysis of existing and planned hydrocarbon activities and infrastructure, overcoming the lack of information that typically hampers large-scale impact analysis. Third, we evaluate the planned activities and infrastructure with respect to the best practice guidelines. We show that Loreto is an extremely active hydrocarbon front, highlighted by a number of recent oil and gas discoveries and a sustained government push for increased exploration. Our analyses reveal that the use of technical best practice could minimize future impacts by greatly reducing the amount of required infrastructure such as drilling platforms and access roads. We also document a critical need to consider more fully the ecological and social factors, as the vast majority of planned infrastructure overlaps sensitive areas such as protected areas, indigenous territories, and key ecosystems and watersheds. Lastly, our cost analysis indicates that following best practice does not impose substantially greater costs than conventional practice, and may in fact reduce overall costs. Barriers to the widespread implementation of best practice in the Amazon clearly exist, but our findings show that there can be great benefits to its implementation.

## Introduction

The western Amazon, one of the most biologically and culturally rich regions on Earth [1]–[3], continues to be an active and controversial zone of hydrocarbon exploration and production [4]. Hydrocarbon blocks – geographic areas delimited by national governments for the exploration and production of oil and gas – cover vast swaths of the region, including protected areas and titled indigenous territories [5]. Moreover, international bidding rounds on new oil and gas blocks in Colombia, Ecuador, and Peru confirm that exploration activities continue expanding deeper into the most remote tracts of the western Amazon. The lone exception is Ecuador's Yasuní-ITT Initiative, a novel government proposal that seeks international compensation in exchange for not drilling sizable oil deposits in the core of the megadiverse Yasuní National Park [1], [6].

With governments promoting ever more oil development in the western Amazon, there needs to be greater attention given to minimizing the associated ecological and social risks [7]. Direct impacts include deforestation for access roads, drilling platforms, helipads, and pipeline routes, as well as contamination from spills, leaks and discharges [5]. Indirect effects, which include selective logging, hunting, and deforestation, primarily arise from the human colonization along new access routes [5]. Considerable social conflict, particularly with native communities, may also arise from these direct and indirect impacts [5].

While we strongly support efforts like the Yasuní-ITT Initiative as a potential mechanism to avoid completely the problems of hydrocarbon activities in the Amazon, we also argue for rigorous best practices where projects do move forward. We define a best practice as one that minimizes the environmental impact associated with typical practice, and that has been successfully employed in a commercial oilfield exploration or production project in Latin America.

At least three major obstacles currently impede the advancement of best practice in the western Amazon. First, best practice lacks a precise set of guidelines in applicable regulations. This regulatory gray area allows project proponents to define almost any practice as “best practice,” and often results in typical high-impact practice being approved as best practice in environmental impact studies. Second, the lack of easily accessible and precise data on planned activities and infrastructure makes it difficult for policy makers and civil society to evaluate upcoming projects and push for best practice. Much of the currently available information relates to just the geographic extent of the hydrocarbon blocks, and not the more important planned activities within. Third, questions regarding cost, or assumptions that best practice will impose substantially greater costs, are common and likely deter companies from deviating from conventional practices.

**Figure 1:**
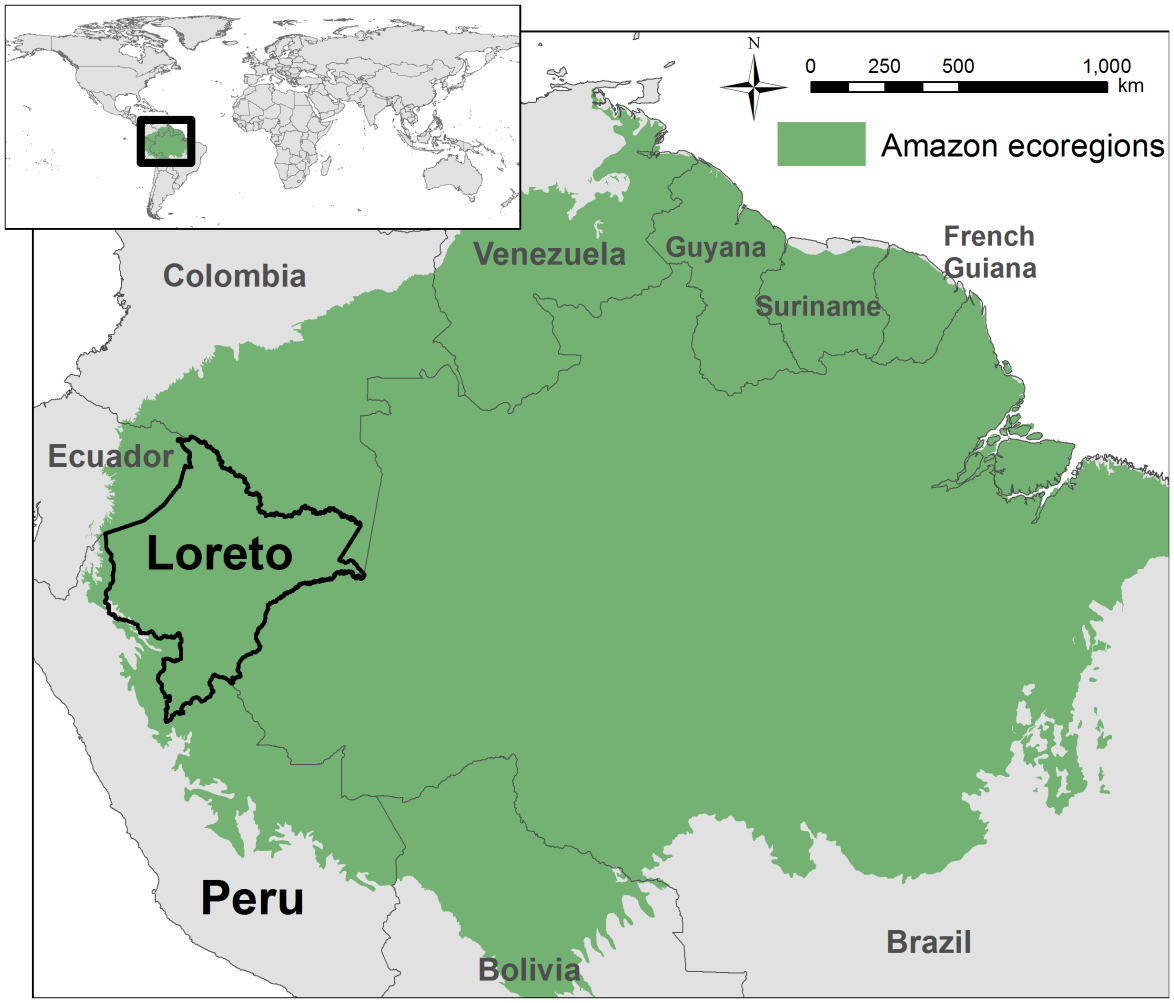
Study focal area. We focus on the Department of Loreto in the northern Peruvian Amazon. Amazon ecoregions are as defined by [61].

We present here a three-part study aimed at overcoming these obstacles and demonstrating the potential of hydrocarbon sector best practice to minimize ecological and social impacts in western Amazonia. Our focus is on the Department of Loreto in northern Peru (Figure 1). Loreto, along with the neighboring Ecuadorian Amazon, is one of the largest and most dynamic hydrocarbon zones in the Amazon [5], [8].

Loreto, a vast territory covering nearly 369,000 km^2^, makes an ideal case study for a number of reasons. The region possesses extraordinary biological and cultural diversity [1], [9], along with vast tracts of largely intact tropical forest, driving an urgency to minimize extractive industry impacts. It is home to a large number of active hydrocarbon blocks spanning the full range of project stages, from pre-exploration to long-time production. In regards to the latter, a pair of 1970s-era oil operations caused significant contamination by dumping toxic production waters into local waterways for nearly four decades [10]. Therefore, local policy makers and residents are acutely aware of the potential risks from oil development. In addition, a number of recent exploration projects have yielded new oil and gas discoveries in Loreto, greatly increasing the probability that hydrocarbon development will continue as a major issue for the region well into the future.

We first present a set of best practice guidelines designed to minimize the impact of hydrocarbon activity in the Amazon. These guidelines incorporate both engineering-based criteria and key ecological and social factors. E-Tech International originally formulated the engineering guidelines, which are based on both Peruvian law and the latest in global technology [11]. We subsequently added the ecological and social factors to ensure that engineering best practice projects also do not threaten sensitive areas.

Second, we provide a detailed analysis of existing and planned hydrocarbon activities and infrastructure. In doing so, we move beyond evaluation based solely on the extent of hydrocarbon blocks and provide a more comprehensive examination of actual activities. This includes detailed data on existing and planned activities for all field-based phases of a hydrocarbon project, namely seismic exploration, exploratory wells, production wells, access roads, and pipelines.

Third, we evaluate the planned activities and infrastructure with respect to the best practice guidelines from part one. We analyze all planned projects in relation to both the engineering guidelines and the following four ecological and social factors: protected areas, indigenous territories, critical ecosystems, and priority watersheds. This evaluation represents a more strategic, larger-scale analysis than the current system of project-level, local-scale studies, and it would ideally take place within the context of a Strategic Environmental Assessment (SEA) [5]. Since 2008, Peruvian law has required national, regional, and local authorities to undertake SEAs for plans, polices, and programs that may have significant environmental impacts [12], [13], but only a handful have been completed to date [14].

We also conduct an initial analysis on the estimated difference in cost between use of best practice and conventional development.

Finally, we discuss our findings in terms of how the use of best practice can minimize negative impacts, particularly deforestation and contamination.

## Results

### Best practice

The basis of the best practice guidelines was an analysis of both cutting-edge technology and Peruvian regulation (Table 1). To understand the implementation of best practice, it is important to understand first the typical life cycle of a hydrocarbon project in the Peruvian Amazon, which follows several basic steps. The government agency Perupetro creates the blocks (“lotes” in Spanish) and then promotes and auctions them internationally [15]. Recently there have been annual or biannual bidding rounds with one to two dozen blocks promoted and auctioned together. Perupetro ultimately signs the final contract with the selected company for each respective block, but the contract must first be approved by presidential decree [15]. The contract term, which runs 30 years for oil and 40 years for natural gas, includes two phases: exploration and production. The exploration phase is for seven years (with possible extensions) and includes a Minimum Work Program for the required amount of seismic lines and exploratory wells to be carried out by the operating company [15].

**Table 1:** Best practice guidelines.

1. Presentation of an overall project development plan based on best practice prior to initiating the exploration phase.
2. Use of state-of-the-art subsurface computer model that integrates airborne electromagnetic data and existing seismic data to minimize the need for new seismic projects.
3. All exploration and production platforms must be capable of drilling Extended Reach Drilling (ERD) wells with a horizontal displacement of at least 8 km (i.e., minimum distance between platforms of 16 km).
4. New access road construction is prohibited (e.g., no new roads between platforms and processing facilities or in pipeline/flowline rights-of-way).
5. Permanent camps may only be constructed along the banks of navigable rivers, not in the jungle interior.
6. Only permissible means of transport are by air and river, with defined limits on the size of transport vessels and on frequency of movements.
7. The maximum pipeline/flowline right-of-way construction width must be less than 13 m with intervals of canopy bridges at least every 1,000 m.
8. Pipelines should be designed/operated with: increased wall thickness to withstand soil movements and effects of internal erosion; regular internal traverses with intelligent inspection tools to detect internal abnormalities and lateral movement of the pipeline; automatic shut-off valves at each tie-in point of welded pipeline sections; and oil spill rapid response teams.
9. Adequate funds must be reserved for site abandonment that includes removal and/or remediation of contaminated materials, soil, and water sources, and revegetation of cleared areas with native species.
10. Consideration of key ecological and social factors such as protected areas, indigenous territories, key ecosystems, and key watersheds in determining whether oil & gas development should be pursued at all.

Two types of seismic testing are common in the Amazon, 2-dimensional (2D) and 3-dimensional (3D) [5], [11]. The former generates an initial 2D cross-section of the subsurface, while the latter generates a 3D model to define in detail the deposit(s). On the ground, 2D is characterized by relatively spread-out linear transects (at least 1 km separation) cut through the forest, whereas 3D lines form tight grids (100s of meters separation) and are typically measured in square kilometers [11]. Seismic lines are typically less than two meters wide and do not require the cutting of large trees. Explosive charges are placed at regular intervals along these lines in holes of six to nine meters, and parallel lines of geophones register the echo patterns of the explosions on subsurface structures. These echo patterns reveal geologic structures that may contain oil or gas and that may warrant further assessment with exploratory wells [11].

If commercially viable quantities of oil or gas are discovered, the concession may proceed to production phase. However, contracts may be, and often are, terminated by the operating company during the exploration phase. Historically, the design of production phase has been characterized by many closely spaced drilling platforms, extensive networks of access roads, and pipeline routes with wide right-of-ways [11]. Moreover, in a number of projects designed during the 1970s, traditional practice included the dumping of toxic production waters directly into local waterways.

#### Engineering criteria

The first step of best practice, from an engineering perspective, is that the operating company must present an overall conceptual plan based on best practice for all phases of the project before beginning any work on the ground. We recommend that such a best practice conceptual plan be required during the company submission of its Minimum Work Program to the government during the bidding phase. This system would have the dual benefit of incorporating best practice into the bidding competition and subsequently the final contract signed by the company and the government. As a result, the use of best practice would be a formal and binding obligation. This recommendation of incorporating best practice into the Minimum Work Program would require a modification to current regulation.

Following this step, exploration activities should combine remote aerial electromagnetic surveys of subsurface structures with existing field information to create a precise state-of-the-art subsurface computer model of the hydrocarbon structures. The construction of this model involves an integrated approach that uses existing field data from seismic testing and exploratory wells as calibration points for new remote sensing data. A recent project in Brazil demonstrated the utility of this integrated approach to produce a precise subsurface computer model with minimal new intervention on the ground [11], [16]. The aim of this innovation is to conduct new seismic testing only in areas where there is a demonstrated potential for commercial deposits. Typically oil companies do not combine the remote sensing data with existing data from earlier exploration programs to refine the study area for the purpose of minimizing the amount of subsequent seismic testing.

At the core of best practice is Extended Reach Drilling (ERD), a technique to reach a larger subsurface area from one surface drilling location. First developed in the late 1980s, ERD is a type of advanced directional drilling where the horizontal reach is at least two times greater than the vertical depth [11]. In practical terms, it means a single drilling platform can reach multiple distant targets in an oil or gas deposit, thereby reducing the total number of required platforms. The U.S. National Petroleum Council [17] recently recognized ERD as a key technology for reducing footprints of drilling operations. The current world record for ERD is 12.4 km, and any horizontal distance up to 8 km is now considered routine for an ERD well [11]. Therefore, there should be a large separation, at least 16 km, between drill sites.

ERD has been used in numerous Latin American exploratory and production drilling projects, but not yet in the Peruvian Amazon. In Argentina, two recent exploration projects employed ERD wells with horizontal displacements of approximately 4 and 5 km, in 2007 and 2008 respectively [11]. Also in Argentina, a production project beginning in 1997 drilled a series of ERD wells of more than 10 km. Most recently, in 2011, an exploration project in Colombia employed an ERD well. Although ERD has not yet seen application in Peru, it is important to note that national hydrocarbon regulation does require that drilling sites disturb the least amount of land possible [18] (see Article 67). Use of ERD would minimize the amount of land disturbed for drilling sites compared to any typical project limited to vertical or directional drilling techniques only.

The use of ERD relates to two additional key best practices: 1) no new access roads, processing facilities, or permanent camps beyond the banks of navigable rivers, and 2) transport of people, materials, and equipment must be by air or river (with controls on size and frequency of movements). In other words, companies must operate as if at sea, a roadless development concept known as the offshore model [19]. In addition, production platforms deeper in the jungle and away from navigable rivers must be unmanned, with raw production fluids transported via roadless flowlines to the respective processing facility located along a navigable river. Processing facilities are where the production fluids – oil, gas, and production water – are separated, and the oil is prepared for export via pipeline, the gas burned for onsite use, and the production water re-injected into a subsurface formation. These points related to roadless development are consistent with Peruvian hydrocarbon regulation, which requires preferential use of river and air transport, and which states that road construction can only proceed if it is demonstrated that river and air transport are not possible [18] (see Article 40). For example, the Camisea natural gas project in southern Peru has been in operation since 2004 with no permanent camps away from navigable rivers and no access roads [11].

Regarding pipelines and flowlines, best practice calls for a greatly minimized right-of-way (ROW), with a reduction from the traditional 25 m down to 13 m or less. This “green pipeline” ROW technique, or “ducto verde” in Spanish, also emphasizes conforming the ROW to natural contours and emphasis on manual clearing (instead of heavy machinery) to further reduce impacts, particularly on steep slopes. This type of reduced-impact pipeline corridor was employed on one ROW section of the Camisea Project, in contrast to the higher-impact traditional pipeline ROWs used in other pipeline/flowline sections of the same project. Another major advantage of this type of narrowed ROW corridor is the ability to maintain canopy bridges. Canopy bridges are tree canopy sections along the ROW that remain intact to facilitate the passage of wildlife, at intervals of approximately one kilometer or more [20]. In order to minimize contamination threats related from pipelines, best practice also calls for increased wall thickness (to withstand soil movements and internal erosion), regular internal traverses with intelligent inspection gauges to detect internal abnormalities and lateral movement of the pipeline, automatic shut-off valves at each welded tie-in point, and establishment of rapid response teams [21].

In terms of site abandonment, companies must set aside adequate funds to assure removal and/or remediation of contaminated materials, soil, and water sources, and revegetation of cleared areas with native species [11].

#### Ecological and social factors

In addition to the engineering-based best practices, it is critical to consider a range of key ecological and social factors. In other words, using technical best practice is not necessarily a license to operate in sensitive areas. Based on previous evaluations of ecological and social factors to consider in assessing projects in areas of high biodiversity and intact forest [5], [8], [22], [23], we chose five: protected areas, priority watersheds, key ecosystems, indigenous territories, and proposed reserves for indigenous peoples in voluntary isolation.

Loreto has 14 official protected areas as established by the national protected areas agency SERNANP. Of these, 11 are managed nationally (two national parks, four national reserves, two communal reserves, and three reserved zones) and 3 are managed regionally (regional conservation areas). In the IUCN system of protected area categories, Peruvian national parks are considered as category II, national reserves as category VI, and the remaining areas either have no category or it is currently undeclared. Of these five types of protected area designations, just national parks are off-limits to extractive industries according to Peruvian Law. However, the new Güeppi – Sekime National Park (established in October 2012) allows the continuation of previously existing concessions. Therefore, 13 of the 14 protected areas in Loreto do not legally prohibit hydrocarbon activities. However, the national protected areas agency (SERNANP) must provide a technical favorable opinion before the energy ministry will approve activities within protected areas.

For priority watersheds, we focus on the Nanay River, a critical resource that provides drinking water to the departmental capital city of Iquitos. The classification of additional priority watersheds in Loreto is still under review by authorities. For key ecosystems, we focus on white-sand forests. Although low in overall species diversity, this rare and fragile ecosystem contains a high number of endemics and is considered a high conservation priority in Loreto [24].

Loreto is also home to a great abundance of indigenous peoples' territories. According to the latest publicly available data from the Instituto del Bien Común (IBC), there are around 500 titled indigenous territories in Loreto. Data for solicited new territories or solicited extensions of existing territories are more preliminary. The IBC data indicate that there are 24 solicited new territories and 29 solicited extensions of existing territories, although the true figures are likely to be much higher for both. In addition, within Loreto there are five proposed reserves for indigenous peoples in voluntary isolation. The right of indigenous peoples to be consulted in order to obtain their free, prior and informed consent about development decisions that will affect them is established under the International Labor Organization's Convention 169 [25] and the United Nations Declaration on the Rights of Indigenous Peoples [26]. Peru is a signatory of the former and voted in support of the latter. Moreover, Peru promulgated a landmark indigenous consultation law based on ILO 169 in 2011 [27].

Finally, two additional factors to consider, but beyond the scope of this study, are the greenhouse gas emissions and use of royalties from hydrocarbon activities. Regarding the former, carbon emissions arise from project-related forest loss, transportation, and energy generation, and of course the ultimate burning of the extracted hydrocarbons [28]. Indeed, one of the selling points of Ecuador's Yasuní-ITT Initiative is not only the avoided on-site deforestation, but also the maintenance of 410 million metric tons of CO_2_ permanently underground [6]. For the latter, it is important to note that over 90% of royalties from hydrocarbon activities go to regional and local governments, and a portion of this money is used for transportation and other development projects that may also have environmental and social impacts [29], [30].

### Existing and planned activities and infrastructure

#### Hydrocarbon Blocks

**Figure 2:**
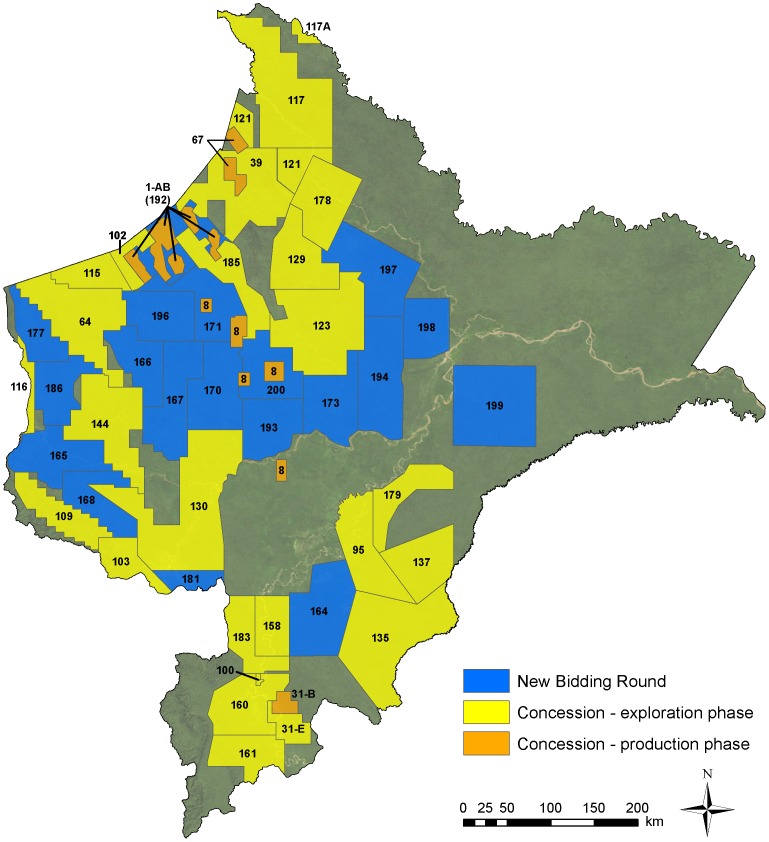
Hydrocarbon blocks in Loreto. There are three general types of blocks based on the contractual agreement between government and a company: concession in exploration phase, concession in production phase, and proposed concession under promotion or negotiation.

As of October 2012, there were 48 hydrocarbon blocks in Loreto (Figure 2), covering 215,169 km^2^ or 57.4% of the department. Of these, 29 are active concessions under contract with multinational energy companies. Four of these active concessions are in production phase (Blocks 1AB, 8, 31B, and 67) and the remaining 25 in exploration phase. The remaining 19 blocks are part of Perupetro's new bidding round.

Of the 25 concessions in the exploration phase, five have approved or pending environmental impact studies for seismic testing, three for exploratory wells, and six for both seismic testing and exploratory wells (Figure S1). The remaining concessions have not yet prepared environmental impact studies or begun exploration work.

Twenty-nine companies were operating or participating in the Loreto concessions during 2012. All but one are multi-nationals based outside of Peru. The 28 multi-nationals originate from 14 countries, including Argentina, Brazil, Canada, Colombia, France, Spain, Vietnam, the United Kingdom, and the United States of America. However, company turnover is relatively high. For example, during the course of this study, the primary concession holder changed in Blocks 64, 67, 123, and 129.

There are two important additional items to emphasize regarding this current state of hydrocarbon blocks in Loreto. First, although at the time of this publication Block 67 was not yet producing oil, the operating company declared this block commercially viable in late 2006, and it is currently officially classified as production phase. Second, many hydrocarbon blocks have previously existed but subsequently been retired and do not appear in Figure 2. Thus, many exploration wells and seismic lines displayed in subsequent figures appear outside the current blocks.

#### Seismic testing

**Figure 3:**
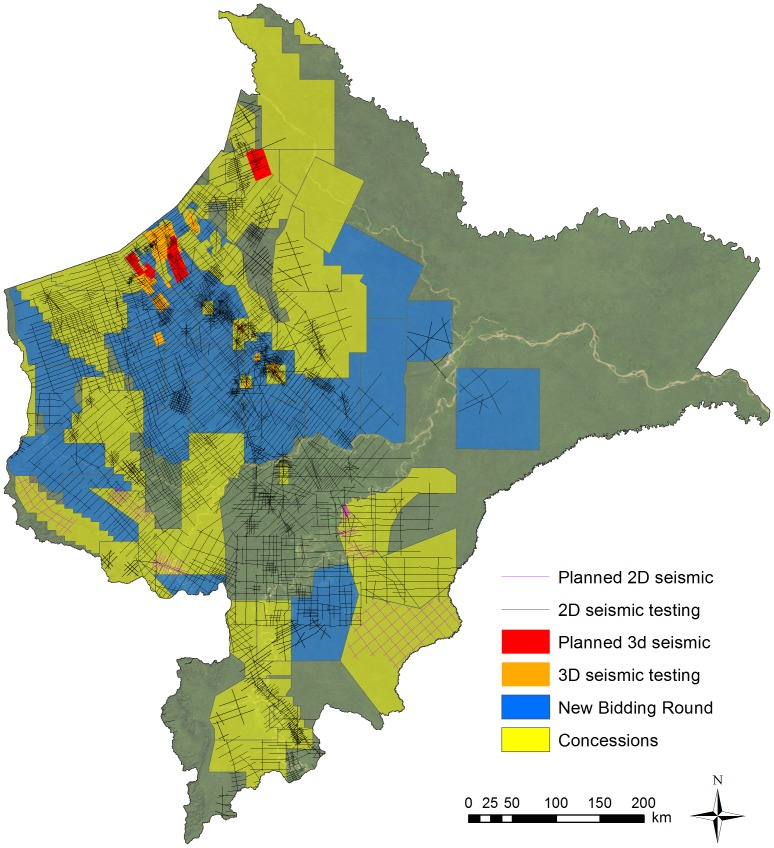
Existing and planned 2D and 3D seismic testing in Loreto. 2D testing is represented by straight lines and is measured in kilometers while 3D testing is represented by polygons and measured in square kilometers. It is important to note that numerous hydrocarbon blocks have previously existed but subsequently been retired. Thus, many seismic lines appear outside the current blocks.

Oil companies have conducted extensive 2D seismic testing in Loreto over the past 40 years, with a smaller but increasing amount of 3D seismic testing in recent years (Figure 3). This includes 61,403 km of 2D seismic lines (9% conducted since 2007) and 2,565 km^2^ of 3D seismic (71% conducted since 2007). As illustrated in Figure 3, testing has been concentrated in southern and central Loreto, while much of northern and eastern Loreto has yet to experience major exploration. In regards to planned testing, five blocks (95, 109, 121, 130, and 135) have pending 2D projects totaling 3,900 km (Figure 3). Two additional blocks (1AB and 39) have pending 3D projects totaling 1,738 km^2^.

#### Exploratory and production wells

**Figure 4:**
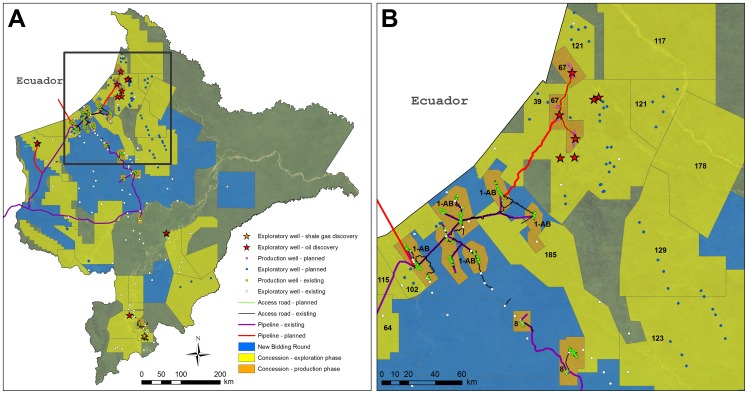
Existing and planned exploratory wells, production wells, access roads, and flowlines/pipelines in Loreto. (A) Map for all of Loreto. Note that stars indicate the Block 64 light crude oil discovery, the Block 95 medium oil discovery, and the Block 31 shale gas discovery. (B) Zoom of high activity zone in and around Blocks 1AB, 39, and 67. Note that stars indicate the Blocks 39 and 67 heavy crude oil discoveries. It is important to note that numerous hydrocarbon blocks have previously existed but subsequently been retired. Thus, many exploration wells appear outside the current blocks.

Official data indicate that oil companies have drilled 223 exploratory wells in Loreto (Figure 4A), with 12% of them drilled since 1998 (the earliest date for which we have detailed data). Of these wells, nearly half (105) are outside of current production blocks and therefore may provide key field information to create subsurface computer models, potentially minimizing the need for extensive new exploratory campaigns.

Companies operating in Loreto have extracted 1.016 billion barrels of oil [31]. Of this production, Blocks 1AB, 8, and 31 have contributed 68%, 31%, and 1%, respectively. Annual oil production in Loreto peaked at 47 million barrels in 1979 [32] and has steadily fallen to 10.2 million barrels in 2011 [31], [33], [34], a decrease of 78%. The Peruvian Energy Ministry estimates over 393 million barrels of oil remain in these blocks (72% in Block 1AB, 25% in Block 8, and 3% in Block 31) [35].

There are currently 219 active production wells in Loreto (Figure 4A). Most are in Block 1AB (62.5%) (Figure 4B), with the remainder in Block 8 (20.5%) and Block 31 (17%). According to the Energy Ministry, there are also ∼50 active reinjection wells and ∼240 inactive and abandoned production wells [31].

Seventeen of the 28 exploratory wells drilled since 1998 have encountered hydrocarbon deposits in Blocks 31E, 39, 64, 67, 95, and 100 (Figure 4A). The type of newly discovered hydrocarbon varies considerably, with light oil in Block 64, medium oil in Block 95, heavy oil in Blocks 39 and 67, and shale gas in Block 31E. The Peruvian Energy Ministry estimates reserves (proven, probable, and possible) of around 928 million barrels in the three blocks with oil (40.5% in Block 39, 35% in Block 67, and 24.5% in Block 64) [35]. An additional 31.6 million barrels is reported from the latest oil discovery in Block 95. In terms of upcoming production, Block 67 is by far the most advanced, with approved environmental impact studies for the pipeline and development wells (Figure 4B).

Environmental impact studies have been submitted for 66 additional exploratory well platforms in Blocks 39, 64, 95, 102, 121, 123, 129, 130, and 135 (Figure 4A).

#### Roads and pipelines

Over time, the companies operating Blocks 1AB and 8 have constructed an extensive access road and flowline network to service the production wells and processing facilities. In addition, the North Peruvian Pipeline transports oil from these blocks to Peru's Pacific coast. Within Loreto, this flowline/pipeline network extends ∼1,156 km (Figure 4A). Transport of crude oil from Block 31 to Pucallpa is via the Ucayali River.

There are plans to extend the existing pipeline network to connect with the new oil discoveries in the region (Figure 4A). The environmental impact study for a new 207 km pipeline to transport heavy crude from Block 67 to the starting point of the existing North Peruvian Pipeline was approved in 2011 (Figure 4B). Completion of this pipeline is scheduled for 2017. Preliminary plans also exist to transport light crude from Block 64 to the existing North Peruvian Pipeline. In August 2012, Peru and Ecuador signed an agreement that would allow the transport of Ecuadorean crude across the border to the North Peruvian Pipeline.

We calculate a cumulative network of 803 km of access roads in Blocks 1AB, 8, and 31 (Figure 4A). The largest access road network by far is in Block 1AB, a sprawling network of 504 km (Figure 4B).

The recently approved environmental impact study for the Block 67 production wells includes plans for a new 85 km access road network adjacent to the internal pipelines during the construction phase (Figure 4B). According to the approved plan, half of this access road network will be eliminated after the construction phase, including the connections between the three oil fields. There are also preliminary plans for construction of a 36 km access road in Block 64 (Figure 4A).

### Best practice

#### Engineering criteria

**Figure 5:**
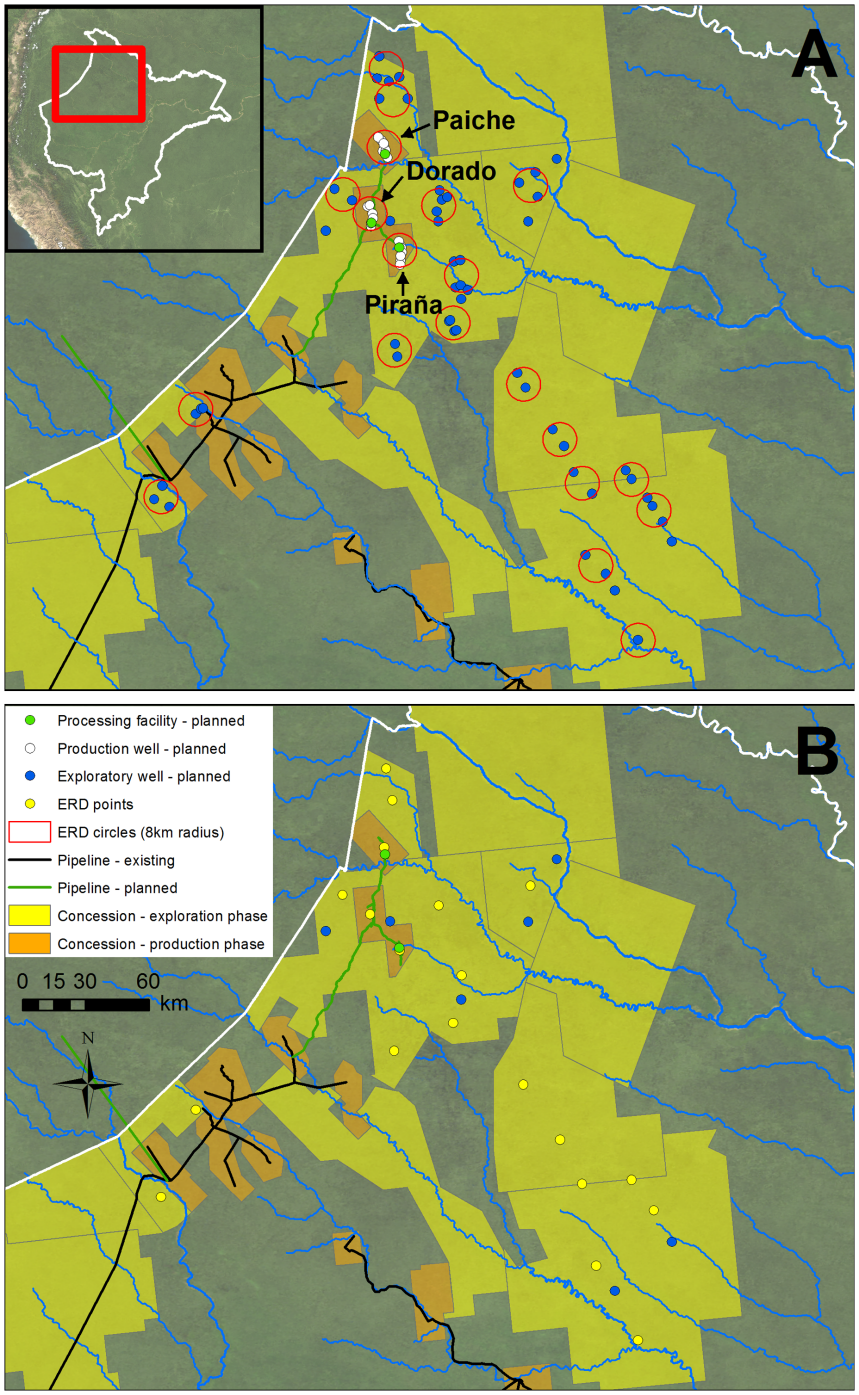
Analysis of planned projects in relation to best practice guidelines in high activity zone of Loreto. (A) Planned exploratory wells, production wells, and processing facilities. Red circles indicate multiple platforms within eight kilometers of a single hypothetical ERD-capable drilling platform. (B) Alternative design based on best practice. Yellow dots indicate where an ERD-capable drilling platform could replace multiple planned platforms. Note that the Dorado processing facility is eliminated due to distance from a navigable river.

We analyzed all planned projects in relation to the best practice guidelines presented earlier. Starting with Block 67 as an example, the production plan consists of 21 production well platforms and three processing facilities (Figure 5a). The platforms are distributed among the three major oil deposits (eight in Paiche, six in Dorado, and seven in Piraña) and each major deposit has its own processing facility. Within each oil deposit, the multiple production platforms are located relatively close together, often separated by less than two km. In each case, the proposed drilling platforms are all within eight km of a single hypothetical ERD-capable drilling platform (Figure 5a). Figure 5b illustrates an alternative ERD-based Block 67 production field design using just three ERD-capable drilling platforms, one for each oil deposit. In addition, note that in Figure 5b, the Dorado processing facility is gone, leaving only the two processing facilities, Paiche and Piraña, located near navigable rivers. This important modification would also mean the elimination of nearly all access roads.

In regards to the planned Block 67 pipeline, the main technical issue is the width of the right-of-way. As noted above, best practice calls for a maximum ROW width of 13 m or less. The Block 67 operating company at the time (Perenco) originally proposed a 25 m ROW width for much of the pipeline length (177 km), with a reduction to 20 m for the 30 km length crossing the Pucacuro National Reserve. Under pressure from the Peruvian government, the company increased the section having a 20 m width to 141 km, but resisted committing to the 13 m width achievable with the green pipeline technique. The company also agreed to a reduced ROW width of 10 m for 0.68 km of the pipeline corridor within Pucacuro that will include canopy bridges.

We also analyzed all other current plans for new exploratory well drilling platforms to determine how many proposed platforms could be eliminated by employing ERD. Figure 5b illustrates an alternative scenario that eliminates all platforms within 16 km of each other, with exploratory wells being drilled up to 8 km from each platform. This scenario assumes each drilling platform is ERD-capable. Of the 66 planned platforms in Blocks 39, 64, 95, 102, 121, 123, 129, 130, and 135, we estimate that nearly half (31) could be eliminated using ERD (Figure S2).

This reduction in infrastructure would translate directly to a reduction in deforestation. According to a sampling of environmental impact studies, we found that each new drilling platform requires the clearing of 2 to 4.5 hectares of forest and production phase processing stations require around 6 hectares each. For example, the Block 67 development project without best practice – consisting of 3 processing stations and 21 drilling platforms – would require a footprint exceeding 1 km^2^ for these facilities. Using best practice to eliminate 18 drilling platforms and one processing facility would reduce forest loss by over 75%. In addition, the new Block 67 access road network and pipeline corridor, without best practice, would result in an additional 7 km^2^ of direct forest loss [36], [37]. With best practice, total direct forest loss would be significantly less, as the vast majority of the roads would be eliminated and the pipeline corridor would be seven to twelve meters narrower along nearly the entire length.

In addition, a review of environmental impact studies and post-project reports reveals that best practice would result in reduced forest loss during the exploration seismic phase. Most seismic projects require at least 50 heliports (larger projects may call for at least 200) and literally hundreds of camps and drop zones [8]. Typical area requirements are around 2,400 m^2^ for helipads, 300 m^2^ for temporary camps, and 20 m^2^ for drop zones. For example, a recently completed 1,480 km 2D seismic operation in Blocks 123 and 129 (that constructed 272 heliports, 208 camps, and 4,050 drop zones) had a cumulative 0.85 km^2^ footprint [38], [39]. A planned 3,700 km 3D seismic operation in Block 39 (calling for 75 heliports, 42 camps, and 3,800 drop zones) projects a 5.99 km^2^ footprint [40].

#### Ecological and social factors

We found that oil blocks overlap 34% (29,000 km^2^) of the protected area system in Loreto, with 19 blocks overlapping 10 protected areas (eight national and two regional) (Figure S3). The protected areas that are the most compromised by oil blocks include the Alto Nanay – Pintuyacu – Chambira Regional Conservation Area, Sierra del Divisor Reserved Zone, and Pucacuro National Reserve. A number of blocks cover an additional 17,150 km^2^ of officially designated protected area buffer zones, primarily around Cordillera Azul National Park and Pacaya-Samiria National Reserve. A number of currently producing wells in Block 8 are within Pacaya-Samiria. Two of the recent Block 39 oil discoveries are within Pucacuro, as is a 30 km stretch of the planned pipeline from Block 67 to the Northern Peru Pipeline. There are 21 planned exploration wells within three protected areas. Thirteen of these planned wells are within Alto Nanay – Pintuyacu – Chambira (Blocks 123 and 129), seven are within Pucacuro (Block 39), and one is within Sierra del Divisor (Block 135).

The vast majority of blocks (90%) overlap titled or petitioned indigenous territories (Figure S4). Put another way, the oil blocks overlap 68% of these indigenous lands (42,548 km^2^). Production wells in Blocks 1AB, 8, and 31 are located around or upstream of indigenous communities. This is also true of the oil discovery in Block 64 and seven additional planned exploratory wells in other blocks.

Twelve oil blocks overlap 60% (21,962 km^2^) of the proposed reserves for indigenous peoples in voluntary isolation (Figure S4). Note that there is an extremely high level of existing and planned activity within the proposed Napo-Tigre Territorial Reserve (Figure S4). The three recently discovered Block 67 oil deposits, two of the Block 39 oil discoveries, and 48 planned exploratory wells are within the reserve. There are also three planned exploration wells in the proposed Yavari-Tapiche Territorial Reserve.

Twelve blocks overlap white-sand forest patches (Figure S5). Indeed, blocks cover all of the known large patches of white-sand forest outside of Allpahuayo-Mishana National Reserve. Several wells in Blocks 123, 129, and 135 are close to white-sand forests. The Northern Peru Pipeline crosses one of the largest white-sand forest patches.

Finally, fourteen planned exploratory wells in Blocks 123 and 129 are within the Nanay watershed, as are sections of four new blocks included in the new bidding round (Figure S6).

**Figure 6:**
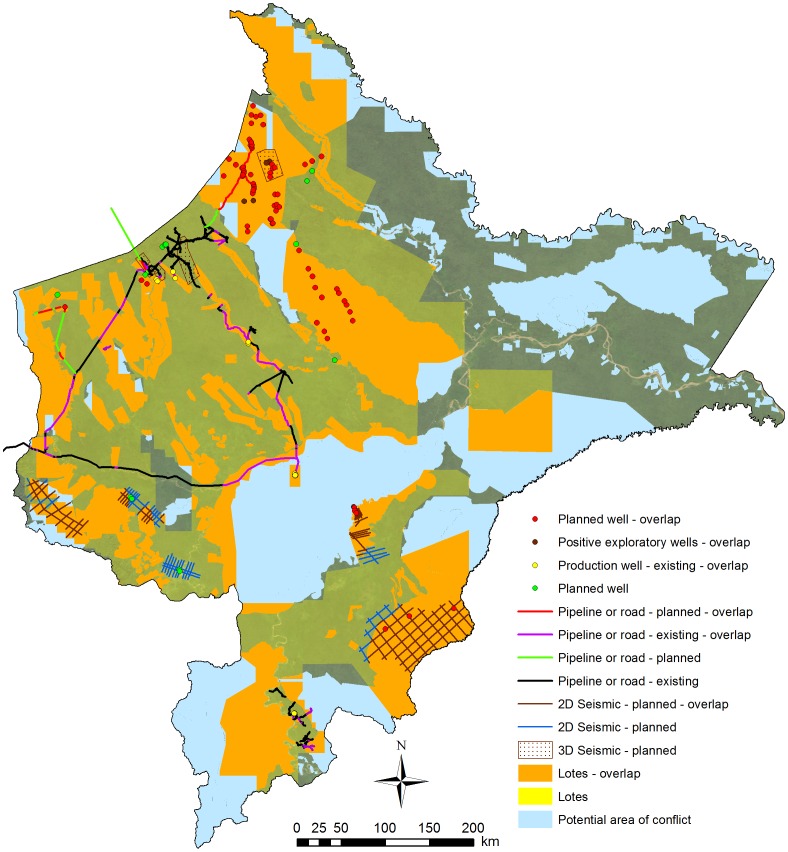
Consideration of key ecological and social factors: overlaps. See Figure S7 for more information on background layer. Light blue indicates an important or sensitive area that is not covered by a hydrocarbon block, while orange indicates an area that is covered by a block. Further, we indicate planned 2D and 3D seismic testing, exploratory and production wells, access roads, and flowlines/pipelines that would overlap with at least one of the key ecological and social factors.

When combining all areas covered by protected areas, indigenous territories, white-sand forests, and the Nanay watershed (Figure S7), we found that nearly half (48%) of the total hydrocarbon block area in Loreto overlaps at least one key ecological or social factor (Figure 6). In addition, 80% of the planned exploratory wells, 100% of the planned production platforms, most of the recent hydrocarbon discoveries, and 59% of the planned pipelines contain such an overlap.

#### Cost analysis

There is enough data available on costs for the planned Block 67 development project to make a comparison between the proposed conventional project and an alternative project using best practice [11], [41]. This cost analysis considered changes due to use of ERD, elimination of the access road network, elimination of one processing facility, and implementation of the green pipeline ROW construction technique.

The average depth of the wells in Block 67 is approximately two kilometers. Therefore, only a well with a horizontal displacement of greater than four kilometers would be considered an ERD well. Assuming a single, central drilling platform in each of the three oil fields, we estimated that one-third of the planned wells would use ERD and the remaining two-thirds would be conventional directional wells. A conventional well costs $3.5 million and the cost of an ERD well increases approximately linearly with its horizontal displacement. Therefore, assuming that an ERD well's average horizontal displacement will be twice that of a conventional well, we estimated an average ERD well cost of $7 million. We calculated that the use of ERD for one-third of the wells would increase costs by about $220 million.

Several other key components of best practice, however, would reduce costs. The elimination of 18 planned drilling platforms due to use of ERD would reduce costs by about $142 million. The elimination of one of the planned processing facilities would reduce costs by about $36.5 million (this estimate includes additional costs for expanding one of the other planned processing facilities to accept more flow).

In terms of transportation costs, the elimination of the access road network would reduce costs by about $45 million. Reliance on extensive jungle road networks and diesel-fueled heavy vehicles, using imported diesel fuel, adds a substantial operational cost. There would be some increase in helicopter flights, though this expense would be offset by the near-elimination of heavy vehicle traffic in the block. In regards to arriving to the site, barges already move on regular schedules from Iquitos to docks of active concessions throughout Loreto and therefore do not represent a major new expense.

Overall, we found that best practice does not translate to substantially higher costs, and may in fact reduce total expenses. The operating company for Block 67 estimated total costs of $1.339 billion [41]. We estimate that total costs for the best practice alternative is $1.321 billion.

## Discussion

Loreto, a vast region larger than Germany or nearly the size of Montana, is one of the most active and dynamic hydrocarbon zones in the Amazon. Forty-eight oil blocks cover over half the department, an affected area of over 215,000 km^2^. These blocks cover the full range of project development stages: 4 in production, 25 in various stages of exploration, and the remaining 19 are part of Perupetro's latest international bidding round. Adding to the complexity, 29 companies operate the production and exploration phase blocks, and company turnover is frequent.

Companies have extracted over one billion barrels of oil from Loreto over the past 40 years. However, a major long-term trend of decreasing production has spurred efforts to boost exploration in search of additional deposits. This trend will begin to reverse with the imminent start of production in Block 67, the most recent block to enter into production phase. Two additional recent notable discoveries include heavy oil in Block 39 and light oil in Block 64. The Peruvian Energy Ministry estimates reserves of over 900 million barrels of oil in these three blocks. Together with the remaining reserves in Blocks 1AB and 8, Loreto may have another billion barrels of oil available.

A key wild card is the shale gas discovery in Block 31E. This discovery is significant because of the potentially large size of the shale formation, the novelty of developing this type of gas deposit in Peru, and the possible utilization of shale fracturing techniques [42]. Recent experience in the United States has demonstrated that there are significant and unique risks associated with shale gas production, and that these risks are not yet fully understood [43].

More new discoveries are likely given that exploration activities remain very active. Indeed, 44 of the 48 blocks in Loreto are in either exploration or bidding phase, 13 of which already have finalized environmental studies for seismic testing and exploratory wells. In other words, extensive and widespread amounts of exploration are still to come.

### Impacts and the role of best practice

With such a large number of hydrocarbon projects, it is critical to advance best practice as a means of minimizing social and environmental impacts in Loreto. The original design and operations of Blocks 1AB and 8 – characterized by many closely-spaced drilling platforms, dumping toxic production waters directly into local waterways, and extensive access road networks – represent high-impact, 1970s-era technology [11]. In contrast, best practice incorporates a number of technological advances and strategic planning techniques to minimize negative impacts, such as deforestation and contamination.

We demonstrated that the use of technical best practice, in the case of Block 67, would reduce impacts by: 1) reducing the number of drilling platforms from twenty-one to three, 2) eliminating one of the three processing facilities, 3) eliminating virtually the entire access road network, and 4) narrowing the pipeline right-of-way. Furthermore, we estimate that the use of ERD-capable drilling rigs across all exploration blocks in Loreto could eliminate about half of the proposed drilling platforms. In the context of a Strategic Environmental Assessment, this would represent a lower-impact, “greener” scenario, in relation to the higher-impact Business-As-Usual scenario.

We further found that this reduction in infrastructure from best practice would directly translate to a reduction in deforestation. In the case of Block 67, forest loss would drop by around 50, 75, and 100% from drilling platforms and processing facilities, the pipeline, and access roads, respectively. Moreover, the reduction of access roads could prevent substantial secondary deforestation. Fortunately, the isolated existing access roads have not yet triggered significant indirect forest loss from subsequent colonization and logging, as roads have in the neighboring Ecuadorian Amazon. If connected to the rest of Peru's road network, as called for in long-term government plans, indirect deforestation would likely quickly escalate.

The reduction in drilling platforms by employing best practice may also serve to reduce contamination. Blocks 1AB and 8 resulted in nearly four decades of significant contamination through the dumping of toxic production waters into local waterways, until indigenous inhabitants forced an accelerated phase-out of this practice between 2006 and 2009 [10], [44]. However, pollution problems continue to plague local communities, as all three current oil producing blocks in Loreto (Blocks 1AB, 8, and 31B) have had major leaks and spills in recent years [45], [46]. In addition to the now mandatory practice of reinjecting toxic production waters, best practice serves to reduce contamination by significantly reducing the number of point sources (i.e., drilling platforms) and designing more strategic flowline/pipeline routes.

Our best practice guidelines also aim to minimize the negative impacts from exploration phase seismic testing. Our review of environmental impact studies and post-project reports revealed that traditional seismic projects do cause deforestation, primarily from the need to construct hundreds of helipads, temporary camps, and drop zones. In addition, seismic testing, particularly the more intensive 3D form, results in helicopter noise, an inux of workers, the cutting of hundreds of kilometers of seismic lines through the understory, and the detonation of thousands of underground seismic charges [47]. A recent study found a significant decrease in the group sizes of the endangered white-bellied spider monkey (*Ateles belzebuth*) during 2D seismic testing in Block 39 [48], although these same researchers found no negative impacts on ocelots (*Leopardus pardalis*) [49].

As part of best practice, we contend that the extent of future seismic testing, and therefore its associated impacts, could be greatly reduced by combining existing exploration data with remote sensing data in a state-of-the-art subsurface computer model. The region has already been subject to over 61,000 km of 2D seismic testing, 2,500 km^2^ of 3D seismic testing, and 220 exploratory wells. However, companies operating in the region typically do not analyze this existing information in combination with remote sensing data for the purpose of minimizing the amount of new seismic testing. Instead, extensive new seismic testing programs are still the norm, as evidenced by the more than 3,400 km of planned 2D seismic and 1,700 km^2^ 3D seismic projects. Given the extensive amount of existing exploration data in Loreto, this modeling advance offers a methodology that may greatly minimize the extent of new seismic campaigns.

We also raised the important need to consider ecological and social factors in addition to technical best practice criteria. We found that nearly half of the total block area and the vast majority of planned exploration wells, production platforms, and planned pipeline length overlap sensitive areas in Loreto. For example, oil blocks overlap over one-third of the protected area system, two-thirds of the titled and solicited indigenous territories, nearly all of the large white-sand forest patches, and nearly the entire Nanay watershed. Recognizing and minimizing these types of conflictive overlaps early in the government's concession evaluation process could avoid future conflicts. For example, the current controversy over planned exploratory wells in the Nanay watershed, the source of the capital city's water supply, could have been avoided by excluding this area from concessions in the first place. However, history may be doomed to repeat itself as four of the new bidding round blocks overlap this same watershed.

Identifying overlaps and possible conflicts with indigenous communities is also an important element of the new indigenous consultation law. This law, which entered into force in April 2012, is debuting in Loreto with the re-leasing of Block 1AB as Block 192 (current contract expires in 2015). Indigenous organizations are demanding a number of important actions, such as the remediation of existing environmental damages, resolution of land-titling disputes, and consultation with affected indigenous communities before the bidding process begins [50]. They are also calling for the elaboration of a Strategic Environmental Assessment for all planned and existing blocks.

Finally, we demonstrated that incorporating best practice does not impose substantially greater costs than a conventional project, and may in fact reduce overall costs. Although costs for ERD wells are around double that of conventional wells, the reduction in costs from elimination of drilling platforms, access roads, and remote processing facilities counterbalance the higher well construction costs.

Large barriers to the widespread implementation of best practice in Loreto and the rest of the Amazon clearly exist. Despite meetings and letters urging Peruvian officials to mandate use of ERD and green pipeline ROW in Block 67, the environmental impact studies were approved without full adoption of these key elements of best practice. Further work is needed to advance the concepts discussed in this paper, ideally in the form of a government-led Strategic Environmental Assessment.

## Methods

We obtained all GIS data described below from existing sources, no field work was conducted in this study. However, in some cases we revised the data if obvious differences were observed in satellite imagery.

### Analysis of existing and planned activities and infrastructure

We obtained GIS data for hydrocarbon blocks, seismic lines, exploratory wells, and pipelines from Perupetro in November 2011 and October 2012. We acquired GIS data for production wells from Perupetro in July 2012. Additional information on seismic testing, exploratory wells, production wells, oil production, and operating companies is from monthly “Informe Estadístico” and yearly “Anuario Estadístico” reports available on the Ministerio de Energía y Minas website (http://www.minem.gob.pe). We acquired information on whether or not recent exploratory wells encountered hydrocarbon deposits from a Perupetro presentation [51] and press reports. We updated the status of the blocks using the environmental impact studies published on the Ministerio de Energía y Minas website. Data pertaining to the new bidding round blocks are from information included in a Perupetro presentation [52].

For existing pipelines, additional GIS data are from the Loreto Regional Government. We compared the Petroperu pipeline datasets to recent Landsat and higher resolution satellite imagery in Google Earth and ArcGIS basemaps to produce a revised pipeline layer. This revised layer included route corrections for known pipelines and the addition of spurs visible in the satellite imagery but not included in either of the original datasets.

For existing access roads, we obtained two GIS datasets. The first was from the national government via the Ministerio de Transportes y Communicaciones. The second was from the Loreto Regional Government. We compared both datasets to recent Landsat and higher resolution satellite imagery in Google Earth and ArcGIS basemaps to produce a revised data layer. This revised layer included route corrections and the addition of spurs visible in the satellite imagery but not included in either of the original datasets.

Data for planned seismic lines and exploratory wells are from environmental impact studies published on the Ministerio de Energía y Minas website. Information related to the planned production wells in Block 67 is from the relevant environmental impact study [37]. For planned pipelines, we obtained information from the relevant Block 67 environmental impact studies [36], [37], a public presentation by a Block 64 operating company representative in Iquitos, Peru (June 2012), and press reports regarding the pipeline extension to Ecuador. For planned access roads, information is from the relevant Block 67 environmental impact study [37] and an operating company report detailing development options for Block 64.

The cut-off date for incorporating new data was March 2013.

### Best practice

We analyzed all planned projects in relation to both the engineering guidelines and identified ecological and social factors. For the engineering criteria component, we identified all planned exploratory wells and production platforms that are within eight kilometers of a single central drilling platform. These wells could therefore be drilled from a central drilling platform using an ERD-capable drilling rig. We also identified all river sections with at least 5,000 upstream cells in HydroSHEDS [53], which we used as a proxy for year-round navigability of the river. This data was used to corroborate the feasibility of limiting permanent camps and processing facilities to sites along navigable rivers. For the estimates on avoided deforestation, we collected information on the area required for drilling platforms, processing facilities, and seismic activities from a sampling of current environmental impact studies and post-project reports from Blocks 39, 67, 102, 123, 127, 128, 129, 130 and 135.

For the ecological and social factors component, we analyzed all existing and planned activities and infrastructure in relation to: protected areas, indigenous territories, white-sand forest patches, and the Nanay watershed. Data for protected areas are from SERNANP [54]. Subsequently we digitized three new areas created after the data were obtained from SERNANP. GIS data for indigenous territories are from the Instituto del Bien Común [55]. Data for white sand forest patches are from NatureServe, Field Museum, and published studies [24], [56], [57]. Analyses were done in ArcGIS 10.1.

For the comparative cost analysis, we used oil industry guidelines on the definition of ERD wells of at least 2:1 ratio of horizontal displacement to vertical depth [58] and the relative cost of an ERD well (proportionate to length of well), industry data on the maximum length of oil and natural gas flowlines [59], and a comparative cost estimate of green pipeline and conventional pipeline ROW construction costs [60]. Specific data for the Block 67 case study came from the actual projected costs estimated by the operating company (Perenco) to fully develop Block 67. These costs were presented in an official environmental impact study response by Perenco [41] and approved by the Energy Ministry in January 2012. This document includes details on the cost of all major Block 67 infrastructure elements, including well development, drilling platforms, processing facilities, permanent camps, roads, docks, and logistical bases.

## Supporting Information

Figure S1 **Status of hydrocarbon blocks in Loreto.** Blocks color-coded to indicate phase of activity within.
(TIF)

Figure S2 **Analysis of planned projects in relation to best practice guidelines across Loreto.** Red circles indicate multiple platforms within eight kilometers of a single hypothetical ERD-capable drilling platform. Note that of the 66 planned platforms, we estimate that nearly half could be eliminated using ERD.
(TIF)

Figure S3 **Hydrocarbon blocks, activities, and infrastructure in relation to protected areas of Loreto.**
(TIF)

Figure S4 **Hydrocarbon blocks, activities, and infrastructure in relation to indigenous territories of Loreto.**
(TIF)

Figure S5 **Hydrocarbon blocks, activities, and infrastructure in relation to white-sand forests of Loreto.**
(TIF)

Figure S6 **Hydrocarbon blocks, activities, and infrastructure in relation to the Nanay watershed. Note** that the waters of the Nanay lead to the departmental capital of Iquitos, providing its drinking water.
(TIF)

Figure S7 **Consideration of key ecological and social factors: background layer.** This background layer indicates important and sensitive areas such as protected areas, indigenous territories, and key ecosystems and watersheds. Details are in Figures S3, S4, S5, S6.
(TIF)
