# Supplementary figures and images for: Potential of Best Practice to Reduce Impacts from Oil and Gas Projects in the Amazon
Source: PLoS One. 2013 May 1;8(5):e63022. doi: 10.1371/journal.pone.0063022 (PMC3641117; doi:10.1371/journal.pone.0063022)

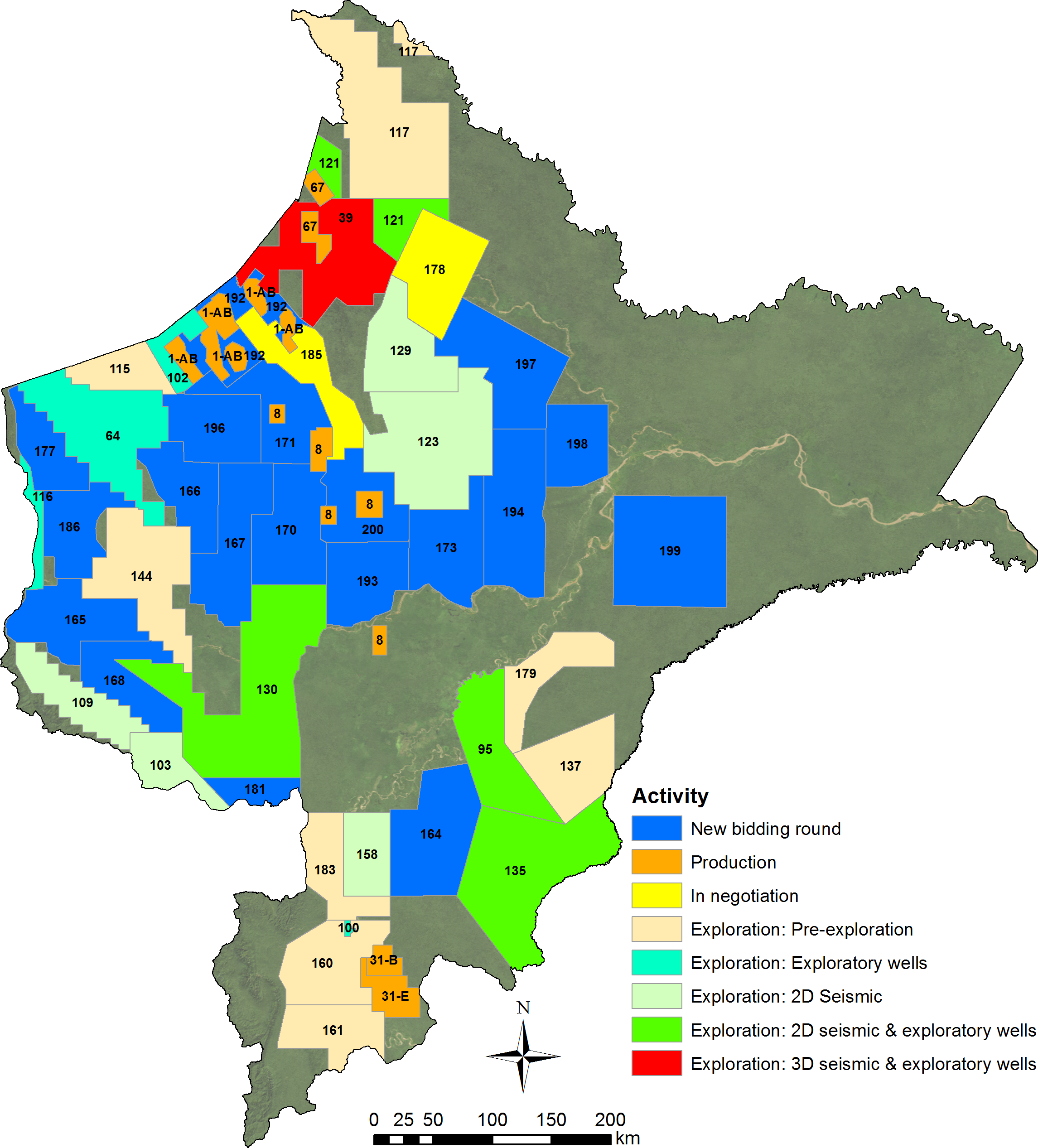

Supplement: Figure S1 — Status of hydrocarbon blocks in Loreto. Blocks color-coded to indicate phase of activity within. (TIF) [file pone.0063022.s001.tif]

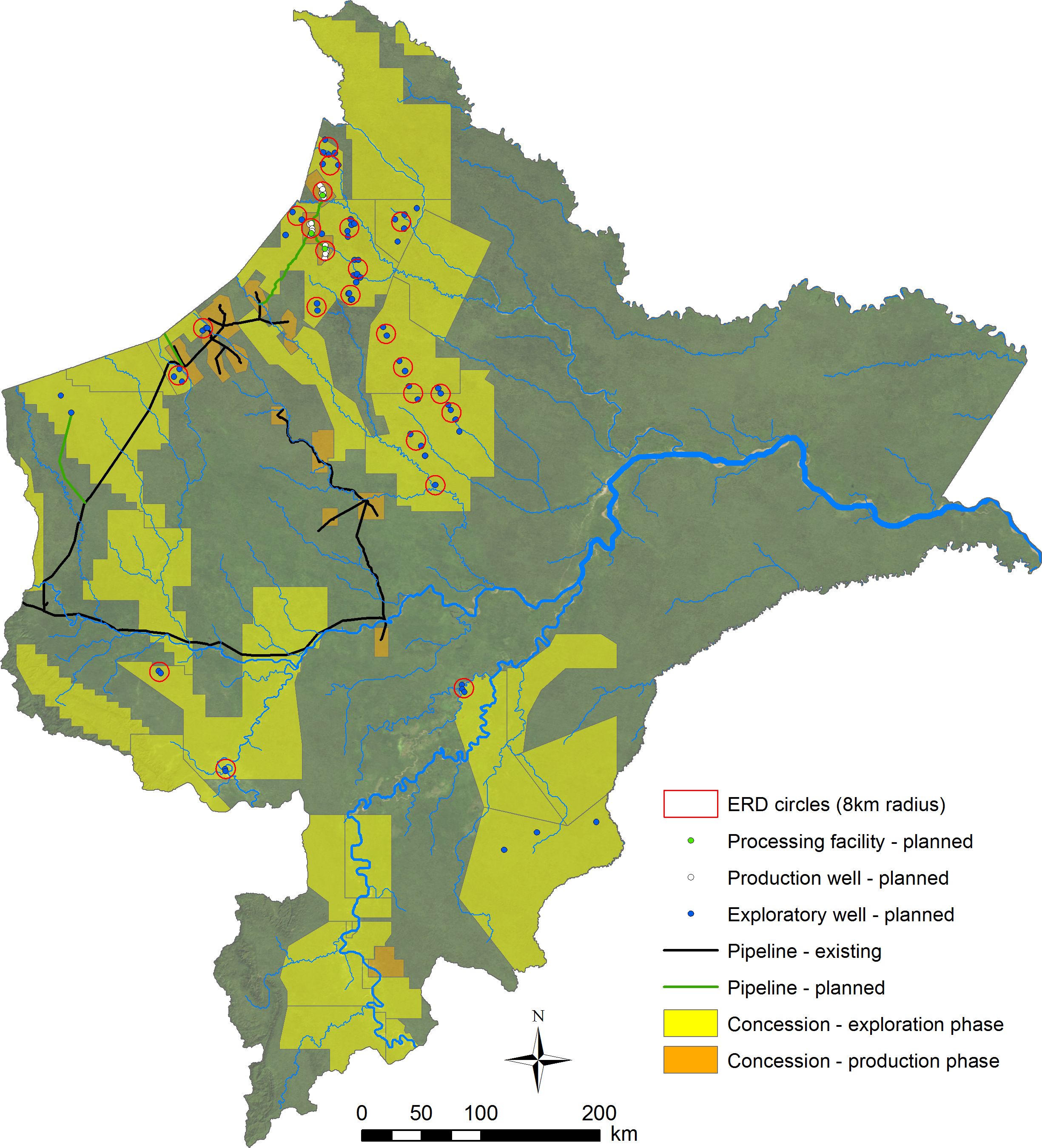

Supplement: Figure S2 — Analysis of planned projects in relation to best practice guidelines across Loreto. Red circles indicate multiple platforms within eight kilometers of a single hypothetical ERD-capable drilling platform. Note that of the 66 planned platforms, we estimate that nearly half could be eliminated using ERD. (TIF) [file pone.0063022.s002.tif]

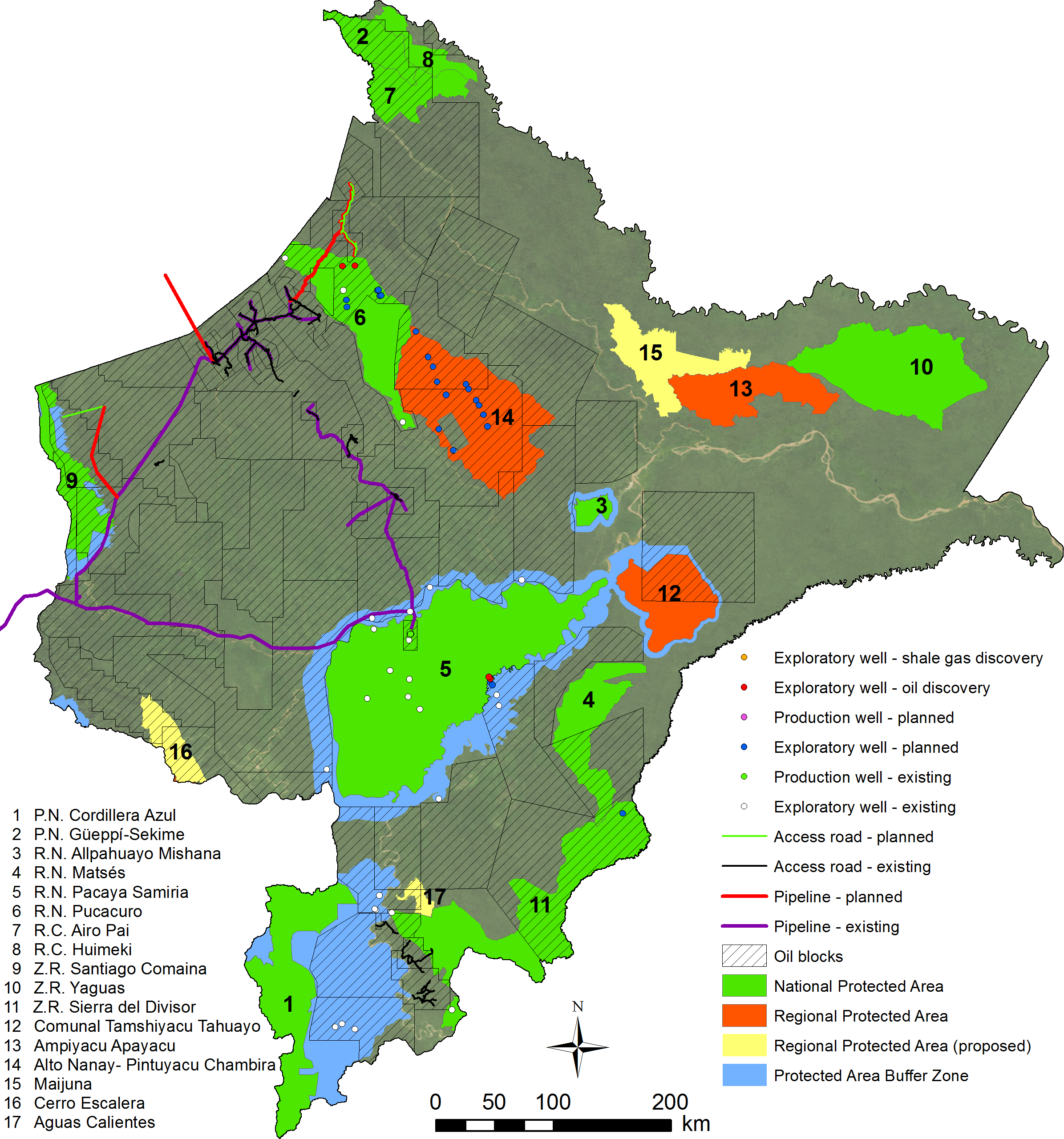

Supplement: Figure S3 — Hydrocarbon blocks, activities, and infrastructure in relation to protected areas of Loreto. (TIF) [file pone.0063022.s003.tif]

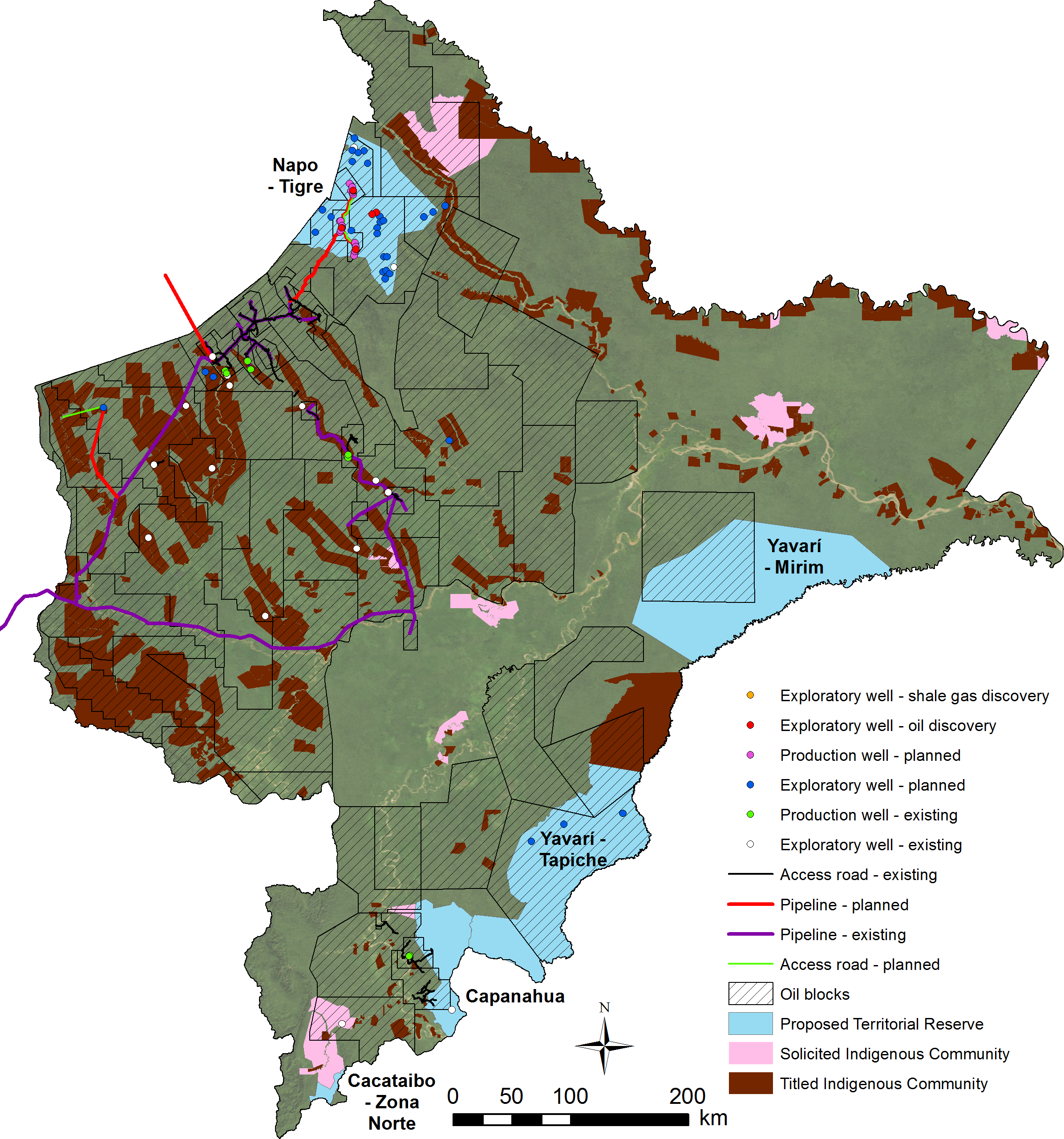

Supplement: Figure S4 — Hydrocarbon blocks, activities, and infrastructure in relation to indigenous territories of Loreto. (TIF) [file pone.0063022.s004.tif]

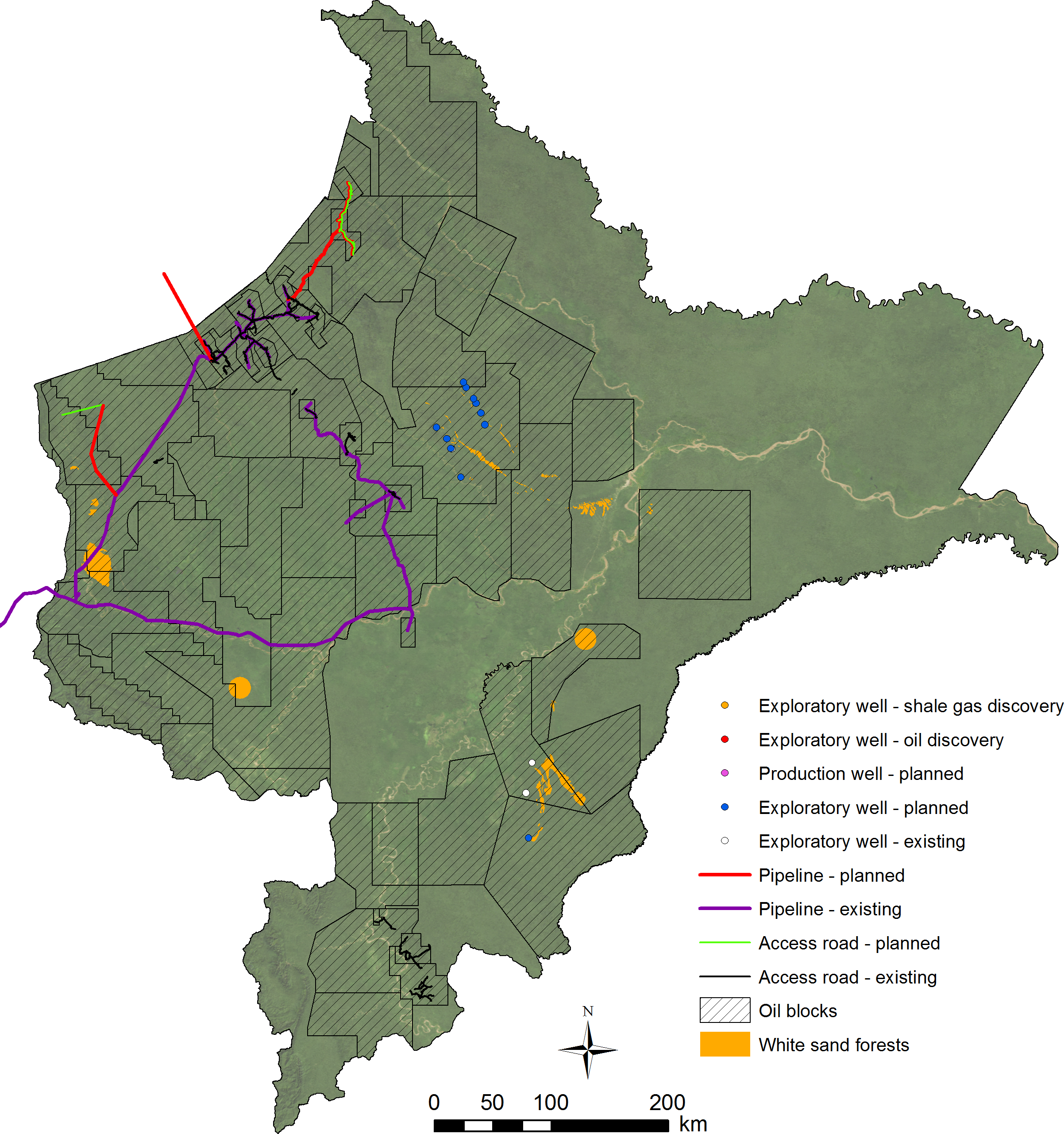

Supplement: Figure S5 — Hydrocarbon blocks, activities, and infrastructure in relation to white-sand forests of Loreto. (TIF) [file pone.0063022.s005.tif]

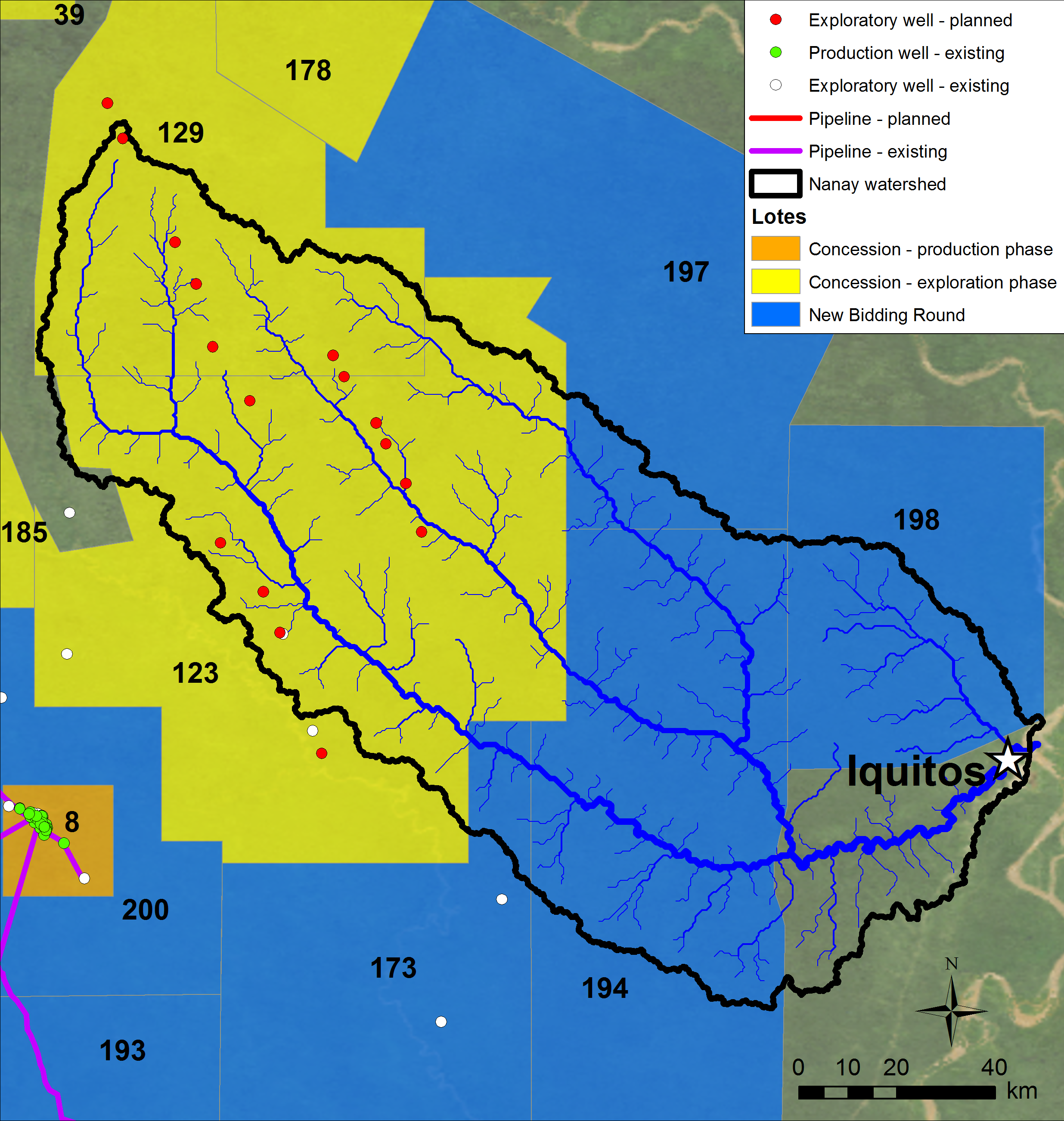

Supplement: Figure S6 — Hydrocarbon blocks, activities, and infrastructure in relation to the Nanay watershed. Note that the waters of the Nanay lead to the departmental capital of Iquitos, providing its drinking water. (TIF) [file pone.0063022.s006.tif]

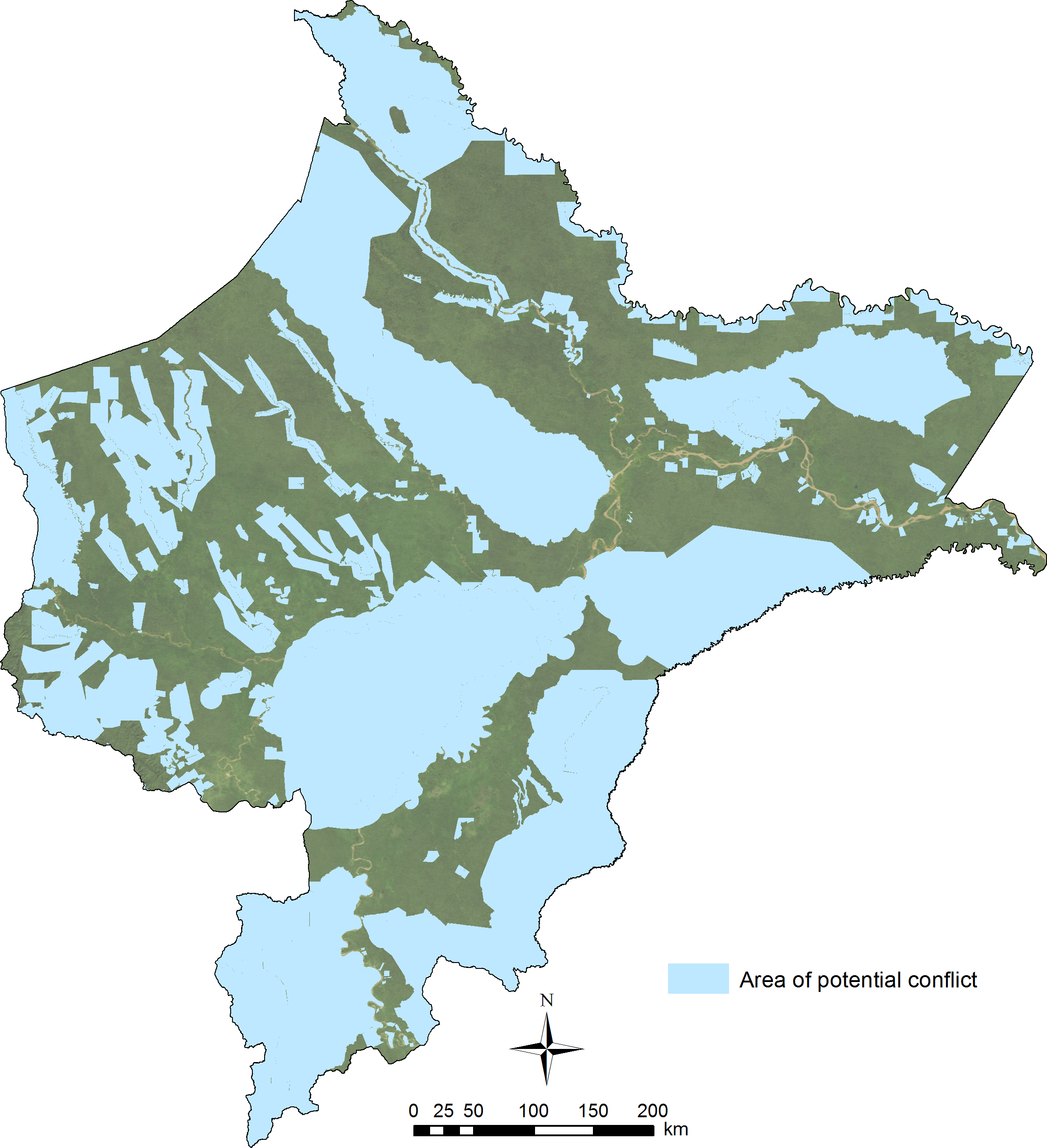

Supplement: Figure S7 — Consideration of key ecological and social factors: background layer. This background layer indicates important and sensitive areas such as protected areas, indigenous territories, and key ecosystems and watersheds. Details are in Figures S3, S4, S5, S6. (TIF) [file pone.0063022.s007.tif]
